# Tuberculosis patients are physically challenged and socially isolated: A mixed methods case-control study of Health Related Quality of Life in Eastern Ethiopia

**DOI:** 10.1371/journal.pone.0204697

**Published:** 2018-10-15

**Authors:** Aklilu Abrham Roba, Tamirat Tesfaye Dasa, Fitsum Weldegebreal, Abyot Asfaw, Habtamu Mitiku, Zelalem Teklemariam, Mahantash Naganuri, Bahubali Jinnappa Geddugol, Frehiwot Mesfin, Hilina Befikadu, Eden Tesfaye

**Affiliations:** 1 Haramaya University, College of Health and Medical Sciences, Harar, Ethiopia; 2 Haramaya University, College of Social Sciences and Humanities, Dire Dawa, Ethiopia; 3 Haramaya University, College of Natural and Computational Sciences, Dire Dawa, Ethiopia; Harvard Medical School, UNITED STATES

## Abstract

**Introduction:**

Pulmonary tuberculosis (TB) impairs respiratory physiology and functional ability, resulting in economic and social dependence upon others. Patients with tuberculosis especially multi drug resistant (MDR-TB) suffer from social isolation, stigma, lack of support and economic constraints. In Ethiopia, the trend of MDR TB is increasing and becoming a serious public health problem. However, little is known about patients except treatment outcomes, financial burden and psychological distress with serious deficiency of data on Health Related Quality of Life (HRQOL). Hence, the aim of this study was to assess HRQOL of MDR TB patients in comparison with drug sensitive pulmonary TB (DSTB) patients.

**Methods:**

We included 100 cases of MDR and 300 controls with DSTB who were matched by sex. Data were collected using SF- 36v2 TM questionnaire and analysed with SPSS version 20. Independent t-test and conditional logistic regression analysis was done considering P-values of less than 0.05 statistically significant. Eight in-depth interviews were also conducted with both groups and represented with verbatim quotations and narrative texts.

**Results:**

There were no statistically significant differences in mean scores for health related quality of life between cases and controls (57.61±16.42 and 59.13±22.10) nor were there significant differences in physical functioning, role disruption due to physical problems, vitality, social functioning, role disruption due to emotional distress, or overall mental health. Individuals with MDR-TB were significantly more likely to be single, a current student, and with lower education and families with more than 5 people than individuals with Drug sensitive TB, all of which were significantly associated with poorer HRQOL (p<0.05). There was good internal consistency of the scale scores, with a Cronbach's alpha value of 0.73.

**Conclusion:**

Individuals with MDR-TB reported statistically worse general health but less bodily pain than individuals with Drug sensitive TB. To regain the role function they lost, we recommend that health facilities, media and all other stakeholders educate the community, households and students about pulmonary tuberculosis, treatment, prevention methods and therapeutic approaches towards TB patients, specifically MDR-TB.

## Introduction

Tuberculosis (TB) is one of the leading causes of infectious disease that resulted in 10.4 million infections and 1.7 million deaths in 2016 [1]. Multidrug-resistant tuberculosis (MDR TB) is a form of TB caused by bacteria that do not respond to the two most powerful first line anti-TB drugs: isoniazid and rifampicin. Ethiopia has one of the highest TB burdens in the world [2,3]. Health Related Quality of Life (HRQOL) is defined as a person's perception of his or her physical and mental health domains, which is then divided into eight sub domains: physical function, physical role, bodily pain, general health, vitality, social functioning, role–emotion and mental health [4].

MDR-TB often leaves long-term sequelae which can have overall unfavourable effects on the physical and social aspects of patients’ HRQOL [5]. Patients’ physical quality of life can be compromised by damage to the lung structure and function [6]. These effects are manifested by abnormalities on chest X-rays and pulmonary function tests even after microbiological cure [6]. The changes hinder the ability to cope with physical activities and exercise tolerance [7].

Patients with MDR TB face extensive psychosocial challenges including hopelessness, stress, stigma and discrimination [8]. These challenges may extend to losing their job when the diagnosis is discovered, decreased marriage prospects, lack of social support and financial burdens [9–15]. The consequences of stigma result in low self-esteem, distress, discrimination, social exclusion and isolation which in turn lead to a decreased HRQOL, non-disclosure and challenges with treatment adherence [16,17].

Tuberculosis is associated with poor HRQOL. Literatures indicate that mental component scores (MCS) were worsen than physical component Scores (PCS) in both drug sensitive TB (DSTB) as well as MDR-TB patients using SF-36 data collection tool[18–21]. The MCS of HRQOL was rated below 50%[20,21] while PCS below 52%[20,21]. Role limitations due to emotional problems were lowest among scores [22]. On the other hand, a systematic review showed that anti-tuberculosis treatment had a positive effect of improving patients' quality of life; physical health tended to recover more quickly than the mental well-being[23]. Another systematic review identified that psychological well-being and social functioning remained impaired in microbiologically cured patients after treatment [24]. The variables like sex, age, marital status, education, occupation and place of residence were associated with HRQoL scores in different dimensions [22,25].

Although a few studies have assessed the HRQOL of tuberculosis patients in Ethiopia, they were limited by either use of non-standardized data collection tool or focusing on TB co-infection with other disease. One study had assessed the HRQOL of TB patients with adequate sample size but used a single question “How would you rate your health-related quality of life?” and scored from 0 (worst) to 10 (best)”[26]. Another study conducted in Ethiopia had assessed the HRQOL among HIV and HIV/TB co-infected patients using WHOQOL HIV tool and indicated that TB/HIV co-infected patients had a lower quality of life in all domains as compared to HIV infected patients without active TB[27].

The trend of MDR TB is increasing and becoming a serious public health problem in Ethiopia [28–30]. However, little is known about patients with MDR TB in the country except for treatment outcomes [31,32], the financial burden [12] and psychological distress [33]. There is a dearth of information on HRQOL of MDR-TB patients, which may play a critical role in the prevention and control of programs. Hence, the aim of this study was to assess HRQOL of MDR TB patients compared with non MDR pulmonary TB patients by using the SF- 36v2 ^TM^ Health Survey questionnaire [34] in different settings of eastern Ethiopia.

## Materials and methods

### Study area, period and design

A health institution-based case-control study was conducted in eight hospitals and three health centres in eastern Ethiopia including Jugal, Hiwot Fana, Karamara, Dil Chora, Sabian, Haramaya, Deder and Chiro hospitals. The health centres were Number One, Legahare and Amir Nur. The region has urban, semi-urban and rural areas with diverse geo-climactic conditions incorporating low, mid and high lands and also people with diverse lifestyles including farmers, agro-pastoralists, pastoralists and urban dwellers. This case-control study was conducted from February 1, 2017 until July 25, 2017. In-depth interviews were conducted from April 1^st^ until 18th, 2018.

### Population

Four hundred pulmonary TB (MDR and non MDR TB) patients were included in the study. Patients who had been on anti-TB drugs for more than one month and volunteered to participate in the study were included. Those with communication problems or who had completed treatment were excluded. Four MDR TB and four non MDR TB patients were recruited for in-depth interviews after the quantitative data collection was completed, in order to understand the lived experiences of patients regarding physical, psychosocial and financial challenges.

### Sample size and sampling techniques

Sample size was calculated using OpenEpi online software with the following assumptions: 80% power (1 − β), 95% Confidence Interval (CI) and odds ratio = 2.03, the case to control ratio was 1:3 and urban residence as behavioural factor from previous study[35]. The final calculated sample size was 400 patients i.e. cases were 100 MDR-TB patients and controls were 300 non-MDR-TB patients. Cases and controls were matched to sex. 100 MDR and 300 non MDR active pulmonary TB patients that fulfilled the inclusion criteria were selected by simple random sampling techniques. For each case, 3 controls were matched by sex from the same health institution and 60% were male in both groups.

### Data collection

The SF-36v2 ^TM^ Health Survey questionnaire was used to collect the data. Respondents answered questions about their HRQOL over the preceding two weeks [36,37]. The SF-36 assesses and measures eight health concepts: 1) limitations in physical activities because of health problems; 2) limitations in social activities because of physical or emotional problems; 3) limitations in usual role activities because of physical health problems; 4) bodily pain; 5) general mental health (psychological distress and well-being); 6) limitations in usual role activities because of emotional problems; 7) vitality (energy and fatigue); and 8) general health perceptions [38].

Data was collected by twelve Batchelor of Science (BSc) nurses and supervised by six Master of Science (MSc) health professionals trained for this study. All questionnaires were checked daily for completeness, accuracy, clarity, and consistency by the investigators and necessary corrections were made accordingly. Eight in-depth interviews with purposively selected MDR and DSTB patients were recorded by audio recorder using semi-structured interview guides after getting permission and written signed consent to enrich the quantitative findings of emotional and social role.

### Data processing and analysis

Data were entered into Epi Data software version 3.1 and exported to SPSS version 22 for analysis. Descriptive statistics were used to depict the frequency, median, mean and standard deviation. Multivariate conditional logistic regression analysis was carried out to examine the associations between dependent and independent variables. Independent t-test was done to determine if there was any statistically significant difference between the mean scores for the 2 groups. P values of less than 0.05 were considered statistically significant. In-depth interviews were conducted in Amharic and Afan Oromo languages and translated to English, transcribed and a thematic framework was developed for summary of data. When possible, we draw on quotes or details from the in-depth interviews to help explain or substantiate the quantitative findings.

### Ethical considerations

Ethical clearance was obtained from the Institutional Health Research and Ethics Review Committee of Haramaya University, College of Health and Medical Sciences. Participants were informed about the purpose, benefits, risks, autonomy to participate and the right to withdraw or refuse to participate in the study at any time. Throughout the study period, confidentiality of the data was strictly followed. Informed written and signed consent was obtained from all participants after the participant information sheet was read and/or they read it in their own language as well as informed, written and signed assent was obtained from children under 18 years of age in addition to consent from parents.

## Results

### Socio-demographics characteristics

A total of 400 study participants, 100 cases and 300 controls, were included in this study, with 100% response rate. Mean age of the participants in the case group was 27.4 ±10.5 years while in the control group it was 33.7 ±13.7 years with a range of 17–74 years. Males formed 60% of the participants in both groups. Of the cases, 47% had only completed primary education, 53% were single and 27% were students. From the control group, 268 (89.3%) were new cases while 32 (10.7%) were being retreated for TB. There were several notable socio-demographic differences between groups. Individuals with MDR-TB were significantly younger (p = .003), more likely to be single (p = .000), less educated (p = .008), current student (p = .018), with a family size greater than 5 (p = .022), and with symptoms for more than 6 months (p = .000) than individuals with DS-TB. (**Table 1**). A total of 8 patients (4 MDR and 4 non MDR pulmonary TB) were successfully interviewed, of which 4 were females. The mean age was 35 years (range of 22 to 50 years).

**Table 1:** Socio-demographic characteristics of participants in the case and control groups attending TB clinics in Eastern Ethiopia from February to July 2017.

Variables		MDR-TB (n = 100) No. (100) %		TB (n = 300) No. (300) %		P-Value
Age	≤24	44	44.0	81	27.0	0.003
	25–34	29	29.0	92	30.7	
	≥35	27	21.0	127	42.3	
Residence	Urban	67	67.0	213	71.0	0.45
	Rural	33	33.0	87	29.0	
Marital status	Married	43	43.0	172	57.3	0.000
	Single	52	53.0	93	31.0	
	Separated / Widowed	5	-	35	11.7	
Educational status	No formal education	26	26.0	75	25.0	0.008
	Primary education	47	47.0	96	32.0	
	Secondary and above	27	27.0	129	43.0	
Occupations	Government Employee	12	12.0	44	14.7	0.018
	Merchant	25	25.0	105	35.0	
	Student	27	27.0	42	14.0	
	Farmer	36	36.0	109	36.3	
Family size	1–2	15	15.0	75	25.0	0.022
	3–5	49	49.0	154	51.3	
	More than 5	36	36.0	71	23.7	
HIV status	HIV positive	14	14.0	41	13.7	0.933
	HIV negative	86	86.0	259	86.3	
TB treatment duration	Less than 6 months	33	33.0	298	99.3	0.00
	Greater than 6 months	67	67.0	2	-	

### Health Related Quality of Life (HRQOL)

Health-related quality of life (HRQOL) was measured using two summary scores: physical and mental health components. The physical health components score measured four domains: physical function, physical role, bodily pain and general health. The mental health components score also measured four domains: vitality, social functioning, emotional role and mental health.

All domain subscales of the instrument’s (SF-36) internal consistency with Cronbach’s alpha were above 0.5. On the overall scale, the Cronbach’s alpha coefficient was 0.73. The two domains with the lowest mean scores in both groups were role limitations due to physical problems, followed by functional impairment due to emotional distress. The general health domain was rated the highest among domains, followed by social functioning and physical function in both groups.

There were no statistically significant differences in mean scores for HRQOL between groups nor were there significant differences in physical functioning, role disruption due to physical problems, vitality, social functioning, role disruption due to emotional distress, or overall mental health. Individuals with MDR-TB reported statistically worse general health (p = 0.02) but less bodily pain (p = 0.05) than individuals with drug-susceptible TB. (**Table 2**).

**Table 2:** Comparison of the health related quality of life domains between MDR and non MDR TB patients eastern Ethiopia from February to July 2017.

Variables	MDR-TB (Case) TB(Control) (n = 100) (n = 300) Mean (SD) Mean (SD)		95% CI		P-value
			Lower	Upper	
Physical function	65 (22.9)	64.65(28.1)	-5.75	6.45	0.91
Physical role	30 (42.2)	35.58 (43.0)	-15.30	4.14	0.29
Bodily pain	61.70(27.27)	67.71(27.55)	-12.25	0.23	0.05
General health	77.40 (16.81)	72.11 (20.23)	1.88	9.71	0.02
Vitality	59.70 (17.88)	55.93 (19.13)	-0.51	8.04	0.08
Social Functioning	69.60 (25.9)	72.2 (25.94)	-8.50	3.28	0.38
Emotional role	34.33 (45.1)	39.8 (44.6)	-15.59	4.70	0.29
Mental health	63.14 (16.7)	65.11 (18.7)	-6.10	2.17	0.35
**SF- summary of components**					
Physical health component score	58.53(15.71)	60.01(23.94)	-6.37	3.25	0.52
Mental health component score	56.69 (19.35)	58.26(21.75)	-6.52	3.55	0.56
**Over all QOL**	57.61±16.42	59.13±22.10	-6.25	3.20	0.52

**Fig 1:**
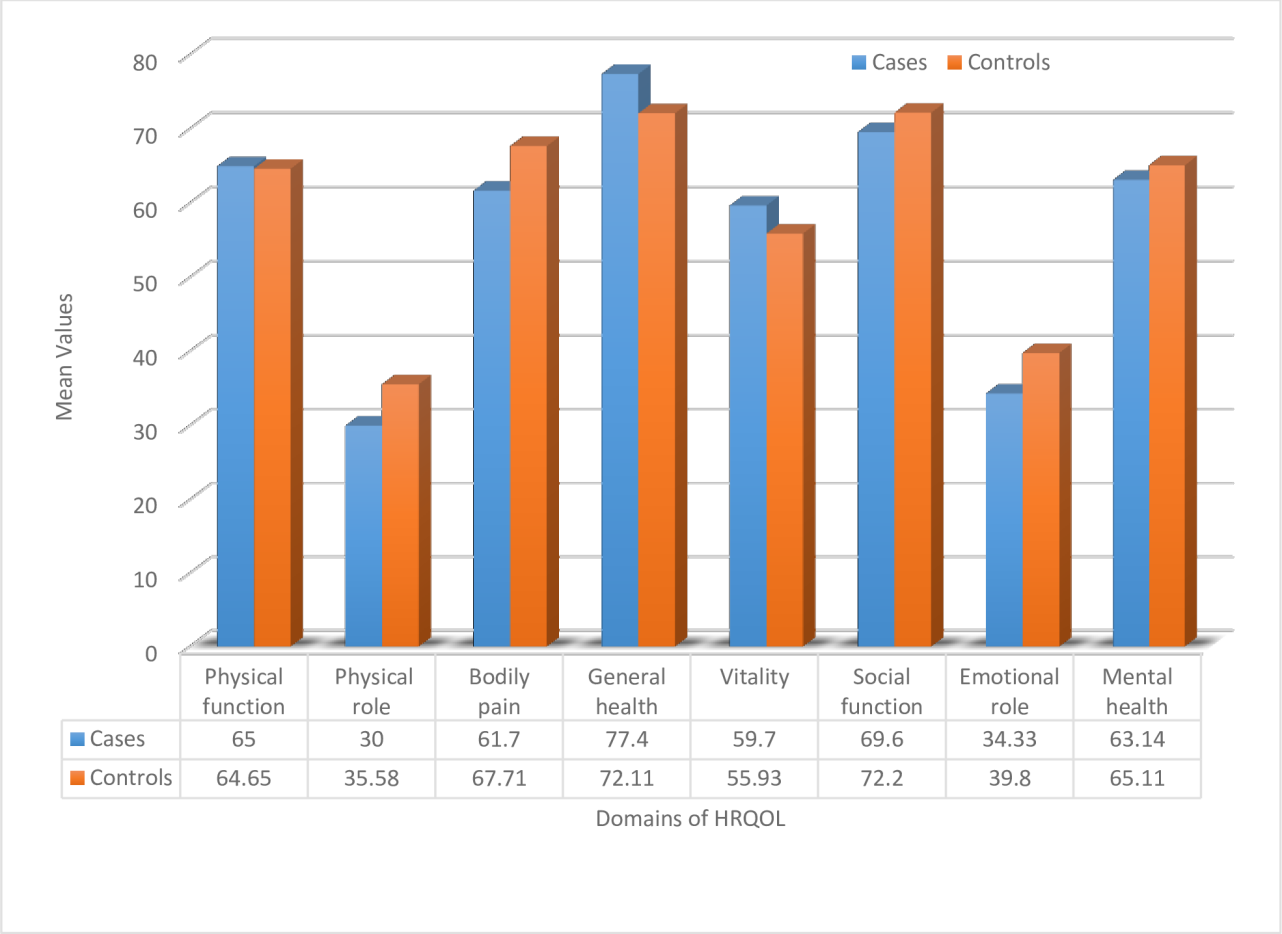
Mean difference of HRQOL domains between case and control groups in eastern Ethiopia from February to July 2017.

The mean differences in scores for the cases were lower than controls in many of the domains as shown in **Fig 1.**

The role limitations due to physical problems and functional impairment due to emotional distress were the lowest from all domains. Similarly, in-depth interviews highlighted that almost all of the patients in both groups had stopped working and faced serious financial burdens due to feeling tired, with the exception of one 38 year-old mother who sold fruits to buy milk to cope side effects of the drugs. All patients mentioned that their physical movement improved with duration on anti-TB treatment. Before or at the beginning of treatment, patients felt severe weakness or tiredness. A female patient, who had worked as a cleaner in private organization, explained the severity of shortness of breath as follows: *“MDR attacks the heart in addition to lungs*, *it suddenly increases my heart rate and I became too tired at the beginning of the treatment*.*”*

Similarly, a college student with MDR TB, who was caring for her mother who had MDR TB and diabetes mellitus (DM) explained that: *“I used to work the whole day and attend my class at night*. *Now*, *I can’t walk for 5 minutes*, *even though I had tremendous improvement being on anti-TB treatment*.*”*

In our study we found that MDR TB patients experienced extensive social stigma and discrimination especially those from rural areas even with strong social and financial positions in the community. The challenges began in their homes and extended to the general community. When a male MDR TB patient, who had been a school director, was asked whether he had any experience of stigma since his diagnosis, he replied: *“uhhh…*.*my wife divorced me*, *all my people ran away including my family*, *my tribe*, *all people who knew me would put their hands on their mouth*. *Really the disease is not as severe as the stigma*. *I have changed 3 rented houses in a 10 month period*. *When the owners of rented houses knew my MDR status (twice I disclosed and once they found out themselves)*, *they threw all my property outside and forced me to leave their house at that minute*. *I was forced not to fulfil my social responsibilities*.*”*

Similar responses were also mentioned by a HIV positive, a farmer and mother of two daughters from a rural region. When she replied to the same question she said: *“when my cough was MDR TB*, *my husband divorced me*. *He forced me to leave the house and I have nowhere to go*. *My neighbours pointed at me*, *and excluded me from social events*. *MDR is more severe than HIV/AIDS*. *My husband supported for HIV but divorced me for MDR*. *When the stigma and discrimination was too severe*, *I moved to a town and was admitted into the MDR centre”*.

However, non MDR TB patients reported relatively better social support from families and communities. When a 50 year-old TB-HIV positive farmer, asked about personal experience of social isolation, she stated: “*my neighbours collect and give me money to buy milk*, *egg and have better nutrition”*. On the other hand, a 54 year old, TB-HIV positive male patient attending his treatment in a hospital explained *“My wife*, *children and older brother give me financial and psychological support to take my medication regularly*.*”*

### Socio-demographic factors associated with HRQOL

In multivariate conditional logistics regression analysis, statistically significant differences between cases and controls for HRQOL were found in certain domains. Poor HRQOL was significantly associated with single marital status (aOR = 1.2, 95% CI 1.32–1.720), being a current student (aOR = 1.4; 95% CI 1.45–2.38), having only a primary school education (aOR = 1.7, 95% CI 1.98–3.46), and having a family size greater than 5 (aOR = 1.3; 95% CI 1.21–1.92 and aOR = 1.6, 95% CI 1.10–2.54). (**Table 3**).

**Table 3:** Socio-demographic factors associated with HRQOL (aOR with 95% CI) in eastern Ethiopia from February to July 2017.

Characteristics		Physical function	Physical role	Bodily pain	General health	Vitality	Social Functioning	Emotional role	Mental health
≤24Years		1	1	1	1	1	1	1	1
25–34 years		1.0(0.7–1.5)	1.0(0.6–1.9)	1.0(0.7–1.5)	0.9(0.6–1.4)	0.9(0.6–1.3)	1.1(0.7–1.7)	0.9(0.5–1.6)	1.1(0.7–1.6)
≥35 years		0.8(0.5–1.3)	0.8(0.4–1.5)	0.8(0.5–1.2)	0.8(0.5–1.2)	0.6(0.4–1.1)	1.0(0.6–1.5)	0.6(0.3–1.2)	0.9(0.6–1.4)
Urban		1	1	1	1	1	1	1	1
Rural		1.1(0.7–1.6)	1.1(0.6–2.2)	1.0(0.7–1.6)	1.1(0.7–1.5)	1.4(0.9–2.1)	1.1(0.7–1.5)	1.1(0.6–2.0)	1.0(0.7–1.5)
Married		1	1	1	1	1	1	1	1
Single		1.3(0.8–1.8)	1.1(0.63–1.84)	1.2(0.8–1.7)	1.2(0.9–1.7)	1.1(0.8–1.7)	1.2(1.3–1.7)	1.1(0.6–1.7)	1.2(0.8–1.7)
Separated		0.8(0.5–1.5)	0.8(0.3–2.1)	0.7(0.4–1.3)	1.0(0.6–1.7)	0.8(0.5–1.6)	0.9(0.5–1.5)	0.8(0.3–2.1)	0.9(0.5–1.6)
No formal education		1	1	1	1	1	1	1	1
Primary education		1.2(0.8–2.9)	1.7(2.0–3.5)*	1.1(0.7–1.7)	1.0(0.7–1.4)	1.2(0.8–1.8)	1.1(0.7–1.5)	1.4(0.7–2.8)	1.1(0.7–1.6)
Secondary and above		0.9(0.6–1.4)	1.1(0.54–2.23)	0.9(0.6–1.4)	0.7(0.5–1.1)	0.9(0.5–1.4)	0.8(0.5–1.2)	1.1(0.5–2.2)	0.8(0.5–1.2)
Gov’t Employee		1	1	1	1	1	1	1	1
Merchant		0.9(0.6–1.4)	0.9(0.5–1.7)	0.8(0.5–1.3)	0.9(0.6–1.4)	0.8(0.5–1.3)	0.9(0.6–1.4)	0.9(0.5–1.7)	0.9(0.6–1.3)
Student		1.2(0.6–2.1)	1.3(0.6–3.1)	1.0(0.5–1.8)	1.1(0.6–1.9)	1.0(0.5–1.8)	1.4(1.45–2.38)*	1.4(0.6–3.2)	1.2(0.6–1.2)
Farmer/Housewife		1.0(0.6–1.6)	0.9(0.4–1.7)	1.1(0.6–1.7)	0.8(0.5–1.3)	0.8(0.5–1.3)	1.0(0.6–1.5)	0.9(0.5–1.8)	0.8(0.5–1.4)
Family size	1–2	1	1	1	1	1	1	1	1
	3–5	1.1(0.8–1.6)	1.0(0.6–1.6)	1.0(0.6–1.4)	1.1(0.8–1.5)	1.4(0.9–2.0)	1.1(0.8–1.6)	0.9(0.5–1.4)	1.0(0.7–1.5)
	>5	1.3(0.8–1.9)	1.0(0.5–1.9)	1.1(0.7–1.7)	1.3(1.2–1.9)*	1.6(1.1–2.5)*	1.1(0.7–1.6)	0.9(0.5–1.6)	1.3(0.8–1.8)
HIV positive		1	1	1	1	1	1	1	1
HIV negative		0.9(0.6–1.3)	1.0(0.5–1.8)	1.0(0.7–1.6)	0.9(0.6–1.3)	0.8(0.5–1.3)	0.8(0.6–1.3)	1.2(0.7–2.2)	0.9(0.6–1.4)

*P < 0.05 **Significant**

## Discussion

Health related quality of life of MDR-TB and DS-TB patients can be contextualized with respect to healthy population for interpreting the average mean scores. We found that some of the scores for MDR-TB and DSTB were 2–3 times lower than healthy population in southern Ethiopia with mean scores of Physical function 93.1 vs 65/64 in this sample; physical role 90 vs. 30/35; bodily pain 90 vs. 61/67; vs General health 72 vs 77/72; Vitality 60 vs 60/56, Social functioning 91 vs 70/72; emotional role 92 vs. 34/40 and Mental health 71 vs 63/65 where the first mean scores denote healthy population, MDR-TB and DS-TB respectively. In other words, although there were very few differences between patients with MDR-TB and DSTB, both had significantly worse HRQOL when compared to the general population in rural Ethiopia[39].

In this study, all eight domains of HRQOL were affected by TB in both MDR and non-MDR TB patients while almost all of the mean scores of the domains for MDR-TB groups were lower than DSTB. This finding was compatible with other studies conducted in Africa and Asian countries [40–43]. We found MDR TB patients had slightly lower mean scores for overall HRQOL than non MDR TB counter parts. This finding was in harmony with other studies conducted in different settings [41,43,44]. This implies that MDR TB patients have low HRQOL even compared to other pulmonary TB patients. However, in our study, the summary scores for physical and mental health components were not significantly different in both groups.

We identified patients with TB have role limitations in social and psychological domains from quantitative data especially among MDR TB patients. In line with this, qualitative data supported the extensive social isolation, discrimination, distress & maltreatment of MDR TB patients. This resulted in an inability to perform social responsibilities, school absenteeism, divorce, unmet spiritual needs, and feeling hopeless with psychological distress. This is comparable with other studies conducted in different regions of the world [8,40,42,45]. Other studies have also indicated that role limitations were associated with self-isolation due to fear of transmission, co-occurrence of TB with HIV/AIDS, lack of awareness about the disease and financial deterioration [9,10,12,46–48].

We found MDR-TB patients from larger family size reported low HRQOL in general health and vitality. This finding is contrary to expectation that having a larger family would be associated with greater social support. Individuals with MDR-TB may have lower quality of life due to isolation as soon as their MDR-TB status was found out and lack even basic cares like food and shelter. The impacts of MDR-TB also strongly affects patient’s psychological wellbeing and social functioning [49]. Tragically, isolation begins from family members and extend to the whole community especially in rural areas despite the role of the individual whether he/she is teacher or daily labourer. This may be due to wrong perception of the community towards MDR-TB disease process, prevention mechanism and treatment options. So, patients lack energy, tired easily, worn out, sick easier and health status worse easier than those who get care and support due to nature of the disease [5,50].

MDR-TB patients reported worse general health but less bodily pain than individuals with DSTB. Less bodily pain for MDR-TB patients may be due to utilization of pain medications and/or pain perception. MDR-TB patients are treated in in-patient basis for the initial long period of time and pain will be also managed by health professionals in the treatment centres while DSTB patients are managed by OPD basis and pain may not be addressed. On the other hand, the intensity of the pain may be the same to both groups but the way they perceive it may differ. For MDR-TB patients life may be more painful than bodily pain as they suffer a lot from financial constraints [9,45], adverse drug side effects [51] and social exclusion. However, MDR-TB patients have worse general health than DSTB. Studies show that MDR-TB patients get sick easily and develop advanced diseases even after micro-biological cure [6]. This results in perceived low general health of MDR-TB patients.

In the final multivariate model, we found that attending only primary education was 1.7 times more likely to have role limitations in the physical domain compared with education at the secondary and higher level (aOR = 1.7, 95% CI = 1.98–3.46). This may be due to the fact that the majority of patients who have only primary education are engaged in labour intensive activities like farming, construction and business that require appropriate lung function as compared to their well-educated counter parts. Unfortunately, following the disease process, residual lesions in the lung impair respiratory function, make patients easily tired and unable to perform their role in the family and community [50,52,53].

We found that students have 1.4 times more deterioration of social role compared to other groups (aOR = 1.4; 95% CI = 1.45–2.38). This may be associated with interrupting their studies and experiencing severe side-effects in MDR-TB groups[54], separate from peers and increased financial burden[55]; self-segregation, guilt, concealment and emotional repercussions[56].

The strength of this study was inclusion of diverse socio-economic and geographic areas that incorporated all MDR-TB treatment centres and having a relatively large sample size. However, some limitations associated with this study include matching for age was not done as well as the study included patients at any stage of treatment rather than specific point in treatment.

## Conclusion

Health related quality of life was low for both MDR-TB and DSTB groups even if they have no statistically significant differences in mean scores. Individuals with MDR-TB reported statistically worse general health but less bodily pain than individuals with DSTB. Individuals with MDR-TB were significantly more likely to be single, a current student, have only primary education and family size of more than 5. To regain the role function they lost, we recommend that health facilities, media and all other stakeholders educate the community, households whether they are large family or single, and students about pulmonary TB, treatment, prevention methods and therapeutic approaches towards TB patients, specifically MDR-TB. We also recommend that there should be future interventional studies related to improving the physical and psychosocial role to enhance HRQOL of TB patients. Patient centered care and therapeutic counselling may contribute more towards meeting the United Nations Sustainable Development Goal 3 (SDG3) target and the End TB strategy of the WHO rather treating only the disease by anti-TB drugs only. Early diagnosis and easy access to MDR-TB treatment is critical to end TB infection and related sequel.

## Supporting information

S1 Table SF-36 Amharic version questionnaire. (DOCX)
