# Supplementary material for: Tuberculosis patients are physically challenged and socially isolated: A mixed methods case-control study of Health Related Quality of Life in Eastern Ethiopia
Source: PLoS One. 2018 Oct 15;13(10):e0204697. doi: 10.1371/journal.pone.0204697 (PMC6188627; doi:10.1371/journal.pone.0204697)
Supplement: S1 Table — (DOCX) [file pone.0204697.s001.docx]

**S1 Table. SF-36 Amharic version questionnaire. (DOCX)**

| ክፍል አን ድ: - የማህበራዊ ሁኔታ የሚዳሰሱ ጥያቄዎች | | |
| --- | --- | --- |
| ጥያቄ | Coding categories | Code |
| 1. ጾታ | 1. ወንድ 2. ሴት |  |
| 2. እድሜ | …………… |  |
| 3. የመኖሪያ ቦታ? | 1. ከተማ 2. ገጠር |  |
| 4. ብሔር? | 1. ኦሮሞ 4. ሱማሌ  2. አማራ 5. ጉራጌ  3. ትግረ 6. ሌላ ካለ……………………….. |  |
| 5. የጋብቻ ሁኔታ | 1. ያገባ /ች 4. የሞተበት/ባት  2. ያላገባ/ች 5. ሌላ ካለ………………………..  3. የፈታ/ች |  |
| 6. ሐይማኖት? | 1. ኦርቶዶክስ 4. ካቶሊክ  2. ሙስሊም 5. ሌላ ካለ ይጥቀሱ…………  3. ፕሮቴስታንት |  |
| 7. የትምህርት ደረጃ | 1. ማንበብ እና መፅሐፍ የማይችል/ት  2. ማንበብ እና መፅሐፍ የሚችል/ት  3. የመጀመራ ደረጃ  4. ሁለተኛ ደረጃ  5. ድፕሎማ ወይም ከዛ በላይ |  |
| 8. ወርሐዊ አማካይ የቤት ገቢ? |  |  |
| 9. የሥራሁኔታ? | 1. የግል ስራ 5. የቤት እመቤት  2. የመንግስት ሰራተኛ 6. ተማሪ  3. ገበሬ 7. የንግድ ስራ  4. መንግስታዊ ያልሆነ ስራ 8. የጉልበት ስራ |  |
| 10. አሁን ያለዎት የቤተሰብ ብዛት ስንት ነዉ? |  |  |
| 11. በትኛዉ ሳንባ ነቀርሳ በሽታ ህክምና ደረጃ ላይ ኖት? | 1. አድስ ሳንባ ነቀርሳ ታካሚ  2. በድጋም ሳንባ ነቀርሳ ታካሚ  3. MDR-TB ታካሚ |  |
| 12. ኤች.አይ..ቪ የምርምራ ዉጤት ምን ይመስላል ? | 1. ቫይረሱ በደሜ ዉስጥ አለ  2. ቫይረስ በደሜ ዉስጥ የለም  3. አላዉቅም |  |
| 13. የሳንባ ነቀርሳ በሽታ ከተያዙ ምን ያህል ጊዜ ሆኖታል | በወር ይጠቀስ |  |
| 14. ሌላ በሀክም የተነገሮት በሽታ ካለ ይቅቀሱ? |  |  |

ክፍ ል ሁሉት : - ለለፉት አራት ሳምንታት ከጤና ጋር ተያያዥ የሆኑ የኑሮ ደረጃን የሚዳስሱ ጥያቄዎች

ከዚህ በታች ለተዘረዘሩት ጥያቄዎች ለእያዳዱ አንድ መልስ ይመልሱ ፡ ፡

100. ጠቅለል አድርገው፤ ሲመለከቱት የጤንነቶ ሁኔታ ምን ይመስላል?

1. እጅግ በጣም ጥሩ ነዉ 4. ለክፉ አይሰጥም

2. በጣም ጥሩ ነዉ 5. መጥፎ ነዉ

3. ጥሩ ነዉ

**101.** ከባለፈዉ አመት ጋር ሲያስተያዩት የአሁኑ ጤና ሁኔታዎ እንዴት ይገለጻል?

1. ከአምና እጅግ በጣም ይሻላል 5. ከአምና እጅግ በጣም ብሶብኛል

2. በተወሰነ መልኩ ይሻላል

3. ተመሳሳይ ነዉ/ለዉጥ የለዉም

4. በተወሰነ መልኩ ከአምና ብሶብኛል

❖ ከዚህ በታች ለተዘረዘሩት ጥያቄዎች በየቀኑ የሚያደርጓቸዉ ናቸዉ ተብሎ ይገመታል፡፡የአሁኑ የጤና ሁኔታህ/ሽ እነዚህን

እንዳታደርጋ(ግያ)ቸዉ ይከለክላል? መልሱ አዎ ከሆነ፣ መጠኑን እንዴት ትገልጸዋለህ/ሽ?

|  | | አዎ በጣም አቅቶኛል | በመጠኑ አቅቶኛል | አይ፣ ያቃተኝ ነገር የለም |
| --- | --- | --- | --- | --- |
| 102. | ከበድ ያሉ እንቂስቃሴዎች ለምሳሌ መሮጥ፣ከባድ እቃወችን ማንሳትና ከበድ  ያሉ ስፖርቶችን መስራት ያቅቶታል? |  |  |  |
| 103. | መጠነኛ ክብደት ያላቸዉን እንቂስቃሴዎች ማድረግ ለምሳሌ፤ ጠረጴዛ/ወንበር/የቤት ቁሳቁስ/ ከቦታ ወደ ቦታ ማንቀሳቀስ እና ቀለል ያሉ ስፖርቶችን ማከናው ያቅቶታልን? |  |  |  |
| 104. | አነስ ያለ ክብደት ያላቸዉን እቃ ማንሳት/መሸከም ለምሳሌ፤ አስቤዛዎችን ፤ ቀለል ያሉ የቤት እቃዎችን ከቦታ ወደ ቦታ ማንቀሳቀስ ያቅቶታል? |  |  |  |
| 105. | ብዙ ደረጃወችን መዉጣት ወይም ተራራማ ቦታዎችን መዉጣት ያቅቶታል? |  |  |  |
| 106. | አንድ ደረጃ መዉጣት ወይም አነስ ያሉ ከፍታዎች መዉጣት ያቅቶታል? |  |  |  |
| 107. | መታጠፍ፣መንበርከክ፣ማጎንበስ ያቅቶታል? |  |  |  |
| 108. | ከሁለት ኪሎ ሜትር በላይ መራመድ(ከህይወት ፋና እስከ ጀጉላ ሆስፕታል)  በእግር መራመድ ያቅቶታል? |  |  |  |
| 109. | ብዙ መንደሮችን በእግሮ መሄድ ያቅቶታል? |  |  |  |
| 110. | ከአንድ መንደር ወደ ለላ አንድ መንደር በእግሮ መሄድ ያቅቶታል? |  |  |  |
| 111. | ራሶን ችለዉ ገላወትን መታጠብ ወይም ልብስ መልበስ ያቅቶታል? |  |  |  |

❖ ባለፉት 4 ሳምንታት ዉስጥ ከዚህ በታች ከተዘረዘሩት ችግሮች ዉሰጥ፤ በአካላዊ ጤና ም ክኒ ያት በሥራዎ ወይም የየእለት

ተግባሮት ላይ ያጋጠሙ ችግሮች አሉ?

112. በስራ ወይም በሌላ እንቅስቃሴ የሚጠቀሙትን ሰዓት ሸራርፎዎል/ቆራርጠዋል? 1. አዎ 2. አይደለም

113. መስራት ከሚፈልጉት በታች ነው ያከናውኑት ? 1. አዎ 2. አይደለም

114. አንዳንድ ሥራወችን እንዳይሰሩ ገድቦታል? 1. አዎ 2. አይደለም

115. ስራዎን መስራት ከብዶታል ወይም ተጨማሪ አቅም ጠይቆታል? 1. አዎ 2. አይደለም

❖ ባለፉት 4 ሳ ምን ታ ት ውስጥ በ ስ ነ -ልቦና ማ ለት ም በድብ ር ት ወ ይን ም በብ ስ ጭት ምክን ያ ት በስራ ቦታ አልያም በተለመደው ለተቀን እንቅሰቃሴ ለይ ከነዚህ ውስጥ ያጋጠሞት ችግር አሉ?

116. በስራ ወይም በሌላ እንቅስቃሴ የምትጠቀመውን ሰአት ቆራርጠዋል? 1. አዎ 2. አይደለም

117. መስራት ከሚፈልጉት በታች ነው ያከናውኑት? 1. አዎ 2. አይደለም

118. ስራዎን እነደሌላ ግዜ በጥንቃቀ አልስሩም? 1. አዎ 2. አይደለም

119. ባለፉት 4 ሳ ምን ታ ት በምን ያህል የ አ ካ ል ወ ይን ም የ ስ ነ-ልቦና ችግሮት በማህበራዊ እንቅሰቃሴ ላይ ከቤተሰቦ፣ ከጎደኛዎ ፣ከጎረቤቶ ወይም ከሌሎች ጋር ባሎት ግንኙነት ላይ ተጽኖ አሳድሮቦታል?

1. ምንም ተጽኖ አላሳደረብኝም

2. በትንሹ

3. በመጠኑ

4. በከፍተኛ ሁኔታ

5. በጣም በከፍተኛ ሁኔታ

120. ባለፉት 4 ሳምንታት ምን ያህል የሰውነት ህመም አጋጥሞታል?

1. ምንም አላጋጠመኝም 5. በከፍተኛ ሁኔታ

2. በጣም በትንሹ 6. በጣም በከፍተኛ ሁኔታ

3. በትንሹ

4. በመጠኑ

121. ባለፉት 4 ሳ ምን ታ ት ዉስጥ በሰዉነቶ ዉስጥ የሚሰማዎ ህመም ሥራዎ ላይ ምን ያህል ተጽኖ ነበረዉ?

1. ምንም ተጽኖ አልነበረዉም 4. በከፍተኛ ሁኔታ

2. በትንሹ 5. በጣም በከፍተኛ ሁኔታ

3. በመጠኑ

❖ ከዚ በታች የተዘረዘሩት ጥያቀዎች ባለፉት 4 ሳምንታት እንዴት ይሰማዎት እና ነገሮች እንደት እንደነበሩ የሚጠይቁ ናቸዉ፡፡ እባኮን ከታች ከተዘረዘሩት አማራጮች መካከል ከአርሶ ሁኔታ ጋር የሚቀራረበዉን አንዱን ይመልሱ

ባለፉት 4 ሳምንታት ምን ያህል ጊዜ

| ተ.  ቁ | ጥያቄ | ሁል ጊዜ | በአብዛኛዉ ጊዜ | በተዎሰነ ጊዜ | አንዳንድ ግዜ | በጣም ትንሽ ጊዜ | በጭራሽ |
| --- | --- | --- | --- | --- | --- | --- | --- |
| 122. | ከመጠን ያለፈ /እጅግ ከፍ ያለ ደስታ ተሰምቶታል ? |  |  |  |  |  |  |
| 123. | ብስጩ ሆነዎ ታዉቃሉ? |  |  |  |  |  |  |
| 124. | ምንም ነገር አይስደስተኝም ብለዉ የበታችኝነት ስሜት ተሰምቶት  ይውቃል? |  |  |  |  |  |  |
| 125. | የተረጋጋና ሰላማዊ ስሜት ተሰምቶት ይውቃል ? |  |  |  |  |  |  |
| 126. | ብዙ አቅም(ጉልበት) አሎት? |  |  |  |  |  |  |
| 127. | ሀዘንና ጭንቀት/የመተከዝ ስሜት ተሰምቶት ይውቃል ? |  |  |  |  |  |  |
| 128. | እንደማይጠቅም ሰው ተሰምቶት ያውቃል |  |  |  |  |  |  |
| 129. | ደስተኛ ሰዉ ነበሩ? |  |  |  |  |  |  |
| 130. | ድካም ተሰምቶት ያውቃል? |  |  |  |  |  |  |

131. ባለፉት 4 ሳ ምን ታ ት ዉስጥ ምን ያህል ጊዜ የ አካላ ዊ ጤና ችግር ወ ይ ም የ ስ ነ-ልቦና መረ በ ሽ በማህበራዊ እንቅስቃሰዎት ላይ

ለምሳሌ በቤተሰብ፣በጓደኞች፣በጎረቤቶት እንድሁም ከለሎች ጋር ያሎት ግኑኝነት ላይ ተጽኖ አድርጎቦታል?

| 1. | ሁል ጊዜ | 4. በጣም ብዙ ጊዜ |
| --- | --- | --- |
| 2. | በአብዛኛዉ ጊዜ | 5. በጭራሽ/ምንም |
| 3. | አንዳንድ ጊዜ |  |

❖ ከዚ በታች የተዘረዘሩት ጥያቀዎች ለእርሶ ምን ያህል እውነት ወይንም ሃሰት ናቸዉ፡፡

| ተ.ቀ | ጥያቄ | በትክክል እውነት | በአብዛኛው እውነት | አላውቅም | በአብዛኛው ሃሰት | በትክክል ሃሰት |
| --- | --- | --- | --- | --- | --- | --- |
| 132. | ከሌላ ሰው ይልቅ በቀላሉ ለበሽታ ተጋላጭ ነኝ |  |  |  |  |  |
| 133. | እንደማንኛዉም ሰዉ ጤነኛ ነኝ |  |  |  |  |  |
| 134 | የጤናዬ ሁኔታ እየተባባሰ እንደሚሄድ እጠብቃለሁ |  |  |  |  |  |
| 135 | ጤናዬ እጅግ በጣም ጥሩ ነዉ |  |  |  |  |  |

መረጃ ሰብሳቢዉ ስም ና ፈርማ

አመሰግናለሁ!!!!!!!!
